# Co-Designing a Green Activity Program With Chinese American People Living With Memory Loss

**DOI:** 10.1093/geroni/igaf122.927

**Published:** 2025-12-31

**Authors:** Jun Luo, Shih-Yin Lin, Jennifer Wong, Lorna Thorpe, Stella Yi, Abraham Brody, Hayley Belli, Rebecca Lassell

**Affiliations:** New York University, New York, New York, United States; New York University Rory Meyers College of Nursing, New York, New York, United States; New York University, New York, New York, United States; New York University, New York, New York, United States; New York University, New York, New York, United States; NYU Rory Meyers College of Nursing, New York, New York, United States; New York University, New York, New York, United States; Indiana University Bloomington, Bloomington, Indiana, United States

## Abstract

Chinese American adults have the highest incidence of Alzheimer’s disease among Asian American groups. Few programs exist to address cognitive decline that are designed with their input. We sought to co-design a nature or ‘Green’ Activity Program (GAP) to promote physical activity tailored to the needs of Chinese American people living with memory loss (PLML) or dementia. Participants were recruited in Brooklyn, New York. Co-design (engaging end-users in design) occurred separately with Chinese American PLML, professional care partners, and outdoor professionals across three 60-90-minute focus groups. Sessions were recorded, transcribed, translated, and analyzed using directed content analysis. A priori Program Design codes were: ‘preferred nature activities,’ ‘session frequency, duration, delivery mode’; Research Design codes were: ‘outcomes,’ ‘recruitment strategies,’ and a ‘referral pathway.’ Participants included Chinese American PLML (n = 4), Chinese American professional care partners (n = 5), and outdoor professionals (n = 6). Program Design preferences were: 1) session duration: 30 minutes, 2) frequency: 4-8 across 12-weeks, and 3) delivery: in-person and virtual. ‘Existing nature activities’ included dance and walking, while ‘preferred nature activities’ included walking, Tai Chi, and Wuxing Cao (5-element exercises). Research Design preferences included ‘outcomes’ of delaying memory loss, improving physical function, and socializing. ‘Recruitment strategies’ included in-person events, with ‘preferred terms’ being “memory loss” and “brain health.” ‘Referral pathways’ involved champions at family health and older adult centers. ‘Barriers’ included physical limitations, inconveniences, and cost. ‘Facilitators’ were a personalized approach. Co-design led to a culturally tailored GAP with/for Chinese American PLML to guide piloting the program in preparation for efficacy testing.

